# Smaller Cerebellar Lobule VIIb is Associated with Tremor Severity in Parkinson’s Disease

**DOI:** 10.1007/s12311-023-01532-6

**Published:** 2023-02-20

**Authors:** Fatemeh Sadeghi, Monika Pötter-Nerger, Kai Grimm, Christian Gerloff, Robert Schulz, Simone Zittel

**Affiliations:** Klinik und Poliklinik für Neurologie, Universitätsklinikum Hamburg-Eppendorf (UKE), Hamburg, Germany

**Keywords:** Parkinson’s disease, Tremor severity, Cerebellum, Volumetry, MDS-UPDRS

## Abstract

Alterations in the cerebellum’s morphology in Parkinson’s disease (PD) point to its pathophysiological involvement in this movement disorder. Such abnormalities have previously been attributed to different PD motor subtypes. The aim of the study was to relate volumes of specific cerebellar lobules to motor symptom severity, in particular tremor (TR), bradykinesia/rigidity (BR), and postural instability and gait disorders (PIGD) in PD. We performed a volumetric analysis based on T1-weighted MRI images of 55 participants with PD (22 females, median age 65 years, Hoehn and Yahr stage 2). Multiple regression models were fitted to investigate associations between volumes of cerebellar lobules with clinical symptom severity based on MDS-Unified Parkinson’s Disease Rating Scale (MDS-UPDRS) part III score and sub-scores for TR, BR, and PIGD; adjusted for age, sex, disease duration, and intercranial volume as cofactors. Smaller volume of lobule VIIb was associated with higher tremor severity (*P* = 0.004). No structure–function relationships were detected for other lobules or other motor symptoms. This distinct structural association denotes the involvement of the cerebellum in PD tremor. Characterizing morphological features of the cerebellum leads to a better understanding of its role in the spectrum of motor symptoms in PD and contributes further to identifying potential biological markers.

**Supplementary Information:**

The online version contains supplementary material available at 10.1007/s12311-023-01532-6.

## Introduction

Increasing evidence point to an important role of the cerebellum in Parkinson’s disease (PD) pathophysiology and the manifestation of clinical hallmarks including tremor, bradykinesia, rigidity, and gait disturbance/postural instability [1, 2]. PD can be classified based on motor features in three subtypes according to MDS-Unified Parkinson`s Disease Rating Scale (MDS-UDPRS) criteria: tremor dominant (TD), postural instability and gait disorders (PIGD), and indeterminate (I) [3]. The heterogeneity of PD motor and non-motor symptoms has led researchers in recent years to consider focusing on the severity of individual symptoms indicated by MDS-UPDRS besides subtype categorization [4–7].

The cerebellum contains somatotopic body representations [8, 9] and has a complex topographical organization, in a way that various connections of cerebellar sub-regions to the cerebral cortex contribute to a range of motor functions [10, 11]. Primary and secondary somatomotor functions are represented in the anterior and posterior lobes of the cerebellum, respectively [12]. In particular, hand movement is represented in ipsilateral lobules III-VI, VIIb, and VIIIa, which send projections to M1 and form a closed-loop circuit.

In light of pathological changes in PD, loss of purkinje cells has been observed in MPTP (1-methyl-4-phenyl-1,2,3,6-tetrahydropyridine) monkeys and mice [13, 14] and there is further evidence of $\alpha$-synuclein-containing lewy bodies deposition in cerebellar molecular layers [15]. Nonetheless, there is inconsistency in terms of PD pathoanatomy and microstructural changes in the cerebellum, particularly in association with disease severity or specific symptoms [2, 16]. Volume reductions in cerebellar white and grey matters as well as cerebellar lobules in correlation with PD disease severity and clinical hallmarks have been reported in several studies [17–20]. The significance of investigating topographical changes in the cerebellum in association with distinct motor function becomes more evident in light of pathologically altered networks in PD. Reciprocal communications between the cerebellum and motor cortex as well as basal ganglia are affected by PD pathology, in particular, due to dopamine degeneration and subsequently dysfunction of dopaminergic pathways [2, 21, 22]. Therefore, structural changes in cerebellar regions with disynaptic projections to the motor cortex and basal ganglia involved in pathological networks are of direct relevance for pathoanatomical investigations of PD motor symptoms [18, 23, 24].

Magnetic resonance imaging (MRI) is a reliable and easily applicable tool in the field of neuroimaging which can be used to delineate fine structures of the cerebellum and correlate them to specific motor symptoms [10]. Recent fully automated MRI tools allow for isolating and segmenting the cerebellum to the scale of lobules from clinical routine T1-weighted images with high accuracy [25–27].

Nevertheless, MRI-based lobular morphology of the cerebellum in PD in relation to distinct motor symptoms is not yet fully explored [11]. Hence, we aimed to find associations between volumes of specific cerebellar lobules and MDS-UPDRS sub-scores representing PD hallmarks of tremor (TR), bradykinesia/rigidity (BR), and PIGD.

## Methods

### Participants

Fifty-five participants with PD who have undergone MRI imaging at the University Medical Center Hamburg-Eppendorf between 2014 and 2017 were retrospectively evaluated for the study. Inclusion criteria were as follows: (1) age between 45 and 80 years, (2) diagnosis of PD according to Parkinson’s Disease UK Brain Bank criteria [28], (3) no history of other neurological disorders (e.g., vascular malformations, ischemic or haemorrhagic stroke, cerebral neoplasia) or major psychiatric illness, (4) high-resolution T1-weighted image available, and (5) written informed consent. The procedure has been approved by the local ethics committee of Hamburg and was conducted in accordance with the Declaration of Helsinki.

Demographic information of the participants with PD comprised of age, sex, disease duration, more affected body-side, and Hoehn and Yahr stage. Motor symptom severity of each participant with PD was evaluated based on MDS-UPDRS part III [29]. The raters were PD nurses that have all been trained by a movement disorder’s expert and experienced neurologist, as well as by training material from the Movement Disorder Society.^1^ More affected body-side was determined by comparing MDS-UPDRS part III scores between the right and left side. Participants with PD were grouped into two subtypes, i.e., tremor dominant (TD) and postural instability and gait disorders (PIGD) using the MDS-UPDRS motor score based on Stebbins et al. [3]. From the results of MDS-UPDRS III, subitems pertaining to the severity of tremor including 3.15 postural tremor, 3.16 kinetic tremor, 3.17 rest tremor, and 3.18 rest constancy were extracted, followed by extracting PIGD subitems including 3.10 gait, 3.11 freezing of gait, and 3.12 postural stability. Then the ratio of the mean tremor scores to the mean PIGD was calculated and used for classification: ≤ 1 indicates PIGD subtype, > 1, and ≤ 1.5 indicates the indeterminate subtype which was used as an exclusion criterion, and ≥ 1.5 indicates TD subtypes. Furthermore, symptom-specific sub-scores were extracted as follows: tremor (TR) score from the sum of MDS-UPDRS items 2.10, 3.15, 3.16, 3.17, and 3.18; bradykinesia-rigidity (BR) score from items 3.3, 3.4, 3.5, 3.6, 3.7, and 3.8; and PIGD score from items 2.12, 2.13, 3.10, 3.11, and 3.12 [30]. These sub-scores were included in statistical regression models to investigate the associations between the corresponding motor symptoms and cerebellar volumes. All scores were calculated in OFF-medication status.

### MRI Analysis

T1-weighted magnetization-prepared rapid gradient echo (MPRAGE) sequences were acquired by a Siemens 3 T Skyra MRI scanner (Siemens Healthcare, Forchheim, Germany) with a 32-channel head coil (echo time (TE) = 2500 ms, repetition time (TR) = 1.9 ms, flip angle = 9°, slice thickness = 1 mm, and voxel resolution = 0.85). MRI imaging was performed within one week after conducting the MDS-UPDRS rating. To stabilize the head position inside the scanner and avoid the occurrence of MRI image artifacts due to head motion (particularly for participants with PD exhibiting tremor), the smallest head coil that would fit the head was used. Furthermore, the head was fixed inside the coil by using stabilizing head cushions. Anonymized and defaced T1-weighted MRI images were visually checked and their origin and orientations were corrected in SPM12 [31]. Then they were automatically processed using the CERES pipeline [27, 32] while providing the age and sex of each subject which were used to create expected bounds for normal development. The volumetry pipeline consisted of the following steps: segmentation, denoising [33], linear registration to the MNI152 template [34], cropping the cerebellum, non-linear registration to the MNI152 cropped templated [34], intensity normalization, and finally subject-specific library non-linear registration and calculation of volumes of 13 cerebellar lobules including lobules I, II, III, IV, V, VI, VIIb, VIIIa, VIIIb, IX, X, crus I and crus II in each cerebellar hemisphere [32, 35, 36], presented as absolute values in cm^3^ [37]. Also, total cerebellar volume and total intracranial volume (ICV) were estimated. Inhomogeneity correction was performed before and after linear registration [38, 39]. The results were visually verified, and no outliers were found.

### Statistical Analysis

In an explorative approach, we fitted separate multiple linear regression models to relate MDS-UPDRS part III as well as TR, BR, and PIGD scores as dependent variables of interest to the total cerebellar volume as well as volumes of individual lobules as independent variables of interest. Since age-related atrophy is evident in the cerebrum and the cerebellum [40], ICV and age were both considered in the models after linear residualization against the specific volumes as described in [41], as well as sex. TR, BR, and PIGD scores were LOG_10_-transformed to improve data distribution. In order to increase the robustness of the findings, a leave-one-out model analysis (LOOA) was equipped to exclude influential points. False discovery rate (FDR) correction was used to correct for multiple comparisons (*n* = 15) across the volumes of interest, separately for the analysis of MDS-UPDRS part III, TR, BR, and PIGD scores. Statistical analyses were carried out using R version 4.0.3 (r-project.org). Statistical significance was assumed at a *P*-value of < 0.05 (corrected).

Roles of specific tremor types, i.e., postural, kinetic, and rest tremor were further explored through a pre-defined post-hoc multiple regression analysis. Each set of tremor scores was extracted from their corresponding items in MDS-UPDRS III (item 3.15 for postural, 3.16 for kinetic, and 3.17 + 3.18 for rest tremor), and the volumetry association with cerebellar lobules was investigated accordingly.

In addition, a post-hoc analysis with focus on the upper extremities, i.e., hand tremor was performed to rule out possible confounding effects of leg and jaw/lips tremor on the outcomes. To this end, the MDS-UPDRS scores representing leg and jaw/lips tremor were subtracted from the tremor score, then the same multiple regression analysis was utilized to investigate the cerebellar volume associations.

## Results

### Demographic and Clinical Data

Table 1 exhibits the demographic and clinical data of the 55 participants with PD (22 female). The cohort had a median age of 65 years with an interquartile range (IQR) of 48–79, median disease duration of 11 years with an IQR of 1–25. The severity of motor symptoms ranged from mild to moderate with a median score of 33, IQR 14–65, and median Hoehn and Yahr stage of 2, IQR 1–4.

**Table 1:** Demographic and clinical data

	*N*	Mean (SD)	Median [Min, Max]
Sex	55		
F	22 (40.0%)		
M	33 (60.0%)		
Age		62.6 (7.60)	65.0 [48.0, 79.0]
Disease duration		11.5 (4.14)	11.0 [1.00, 25.0]
PD subtype			
PIGD	45 (81.8%)		
TD	10 (18.2%)		
MDS-UPDRS total (OFF)		62.6 (20.8)	58.5 [33.0, 121]
MDS-UPDRS III (OFF)		35.5 (13.3)	33.0 [14.0, 65.0]
Hoehn and Yahr (OFF)		2.4 (0.7)	2.00 [1.00, 4.00]
BR Score		20.4 (8.2)	20.0 [5.00, 41.0]
TR Score		6.4 (6.9)	4.00 [0, 26.0]
PIGD Score		6.3 (4.1)	6.00 [0, 15.0]

*MDS-UPDRS*, Movement disorders Society United Parkinson’s disease Rating Scale; *MDS-UPDRS III*, part III motor examination; *BR*, bradykinesia; *TR*, tremor; *PIGD*, postural instability and gait disorders; *SD*, standard deviation

### Volumes of the Cerebellum and Cerebellar Lobules vs. Symptom Severity

**Fig. 1:**
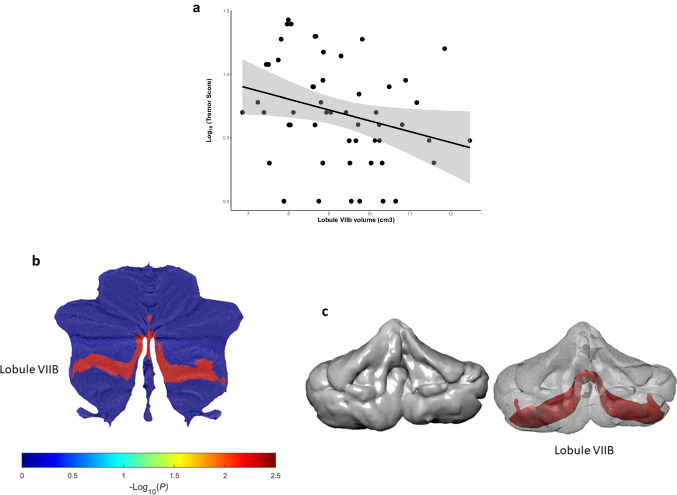
**a** Association between volume of cerebellar lobule VIIb and tremor severity score in PD through linear regression analysis. Note the logarithmic scale on the *y*-axis. Individual lobule VIIb volumes and total tremor scores are included as scatter points in the plot. The grey region surrounding the regression line refers the confidence interval (95%), **b** flatmap of the cerebellum, color bar representing *P*-values corresponding to beta coefficients of lobule volume with total PD tremor score. *P*-values are expressed as -Log10(P) for providing more clarity in visualization, **c** left: three-dimensional reconstruction of the cerebellum, right: bilateral lobule VIIB highlighted

A descriptive overview of cerebellar lobular volumes is provided in table S1. The linear regression models revealed a significant negative association between the volume of lobule VIIb and tremor severity score (*P* = 0.004, Fig. 1, Table 2). No other associations were found between volumes of the total cerebellum or individual lobules with TR severity (Table 2). The linear regression models also revealed a significant contribution of disease duration (*P* = 0.006) to TR score, but neither age, sex nor ICV (Table 3).

**Table 2:** Association between cerebellar volumes and tremor scores in participants with Parkinson’s disease (PD). Results of multiple linear regression models are presented and the primary outcome demonstrated via beta coefficient

Cerebellar Region	Beta (coefficient) (95% CI)	*P*-value
Cerebellum	− 0.01	0.166
Lobule I-II	− 2.70	0.031
Lobule III	− 0.38	0.063
Lobule IV	0.06	0.510
Lobule V	− 0.03	0.546
Lobule VI	< 0.00	0.835
Crus I	0.01	0.690
Crus II	− 0.04	0.094
Lobule VIIb	− 0.13	0.004*
Lobule VIIIa	− 0.05	0.152
Lobule VIIIb	− 0.03	0.385
Lobule IX	− 0.05	0.284
Lobule X	− 0.15	0.502

*P-value significant after leave-one-out (LOOA) analysis as well as FDR correction

The confidence interval is considered as 95%. Cerebellar lobules exhibiting a significant association with tremor severity score are highlighted in bold. Results are adjusted for age, sex, disease duration, and intracranial volume (ICV)

**Table 3:** The winning linear multiple regression model indicating contribution of lobule VIIb volume to tremor severity, as well as age, sex, disease duration, and intracranial volume (ICV) as covariable

Model variables	Beta (coefficient)	*P-*value	95% CI	
			lower	upper
Volume of VIIb (cm^3^)	− 0.13	0.024*	− 0.21	− 0.04
Age (years)	− 0.01	0.339	− 0.02	0.01
Sex (M/F)	0.05	0.289	− 0.20	0.30
Disease Duration (years)	− 0.03	0.006*	− 0.06	− 0.01
ICV (cm^3^)	< 0.01	0.386	0.00	0.00

*P-value significant after leave-one-out (LOOA) analysis as well as FDR correction

Significant factors are highlighted in bold. The primary outcome is reported as beta coefficient and confidence interval is considered as 95%

Models for bradykinesia and rigidity, operationalized by BR and PIGD scores, did not reveal any significant structure-motor symptom relationships (all *P* > 0.13, see tables S2 and S3, respectively).

The post-hoc analysis of different tremor types revealed that kinetic tremor severity was driving the significant association with volume of lobule VIIb surviving both LOOA and FDR correction (*P* = 0.002). In spite of contributing to the main finding, neither postural tremor (FDR corrected *P* = 0.066) nor rest tremor (FDR corrected *P* = 1.000) was found to be associated with lobule VIIb volume (see tables S4-6).

The post-hoc regression analysis focused on hand tremor reproduced the main outcome, yielding a significant correlation between volume of lobule VIIb and the hand tremor score (*P* = 0.005, see table S7).

## Discussion

The results of the present study indicate significant structural associations between cerebellar lobule VIIb volume and the severity of tremor in PD.

Structural changes of the cerebellum in individuals with PD can be associated with decreased resting-state functional connectivity between the cerebellum and sensorimotor network, and importantly the severity of PD tremor can be predicted by combining cerebellar lobules volumetric features and cerebellar-sensorimotor cortex connectivity [23]. The role of cerebellum in the mechanism of PD tremor can be explained through a network perspective. In recent years, the “dimmer switch” model has gained widespread recognition for providing mechanistic insights into PD tremor on a network level while taking all key cortical and subcortical regions of tremor pathophysiology into consideration. According to this framework, the tremor initiates in the basal ganglia (tremor on/off switch) and propagates to the cerebello-thalamo-cortical (CTC) loop (tremor dimmer switch), where the low-frequency oscillation is maintained and even amplified [42, 43]. Besides being a major node in the CTC loop, cerebellum directly communicates with basal ganglia functionality in PD through disynaptic projections [25]. In particular, topographically organized connections exists from the cluster of lobule VII in the cerebellum to not only M1 [24], but also to second-order neurons in the sensorimotor part of the subthalamic nucleus (STN), where substantial input from the primary motor cortex and premotor areas are received [44]. Importantly, STN is the region where pathological tremor oscillations appear, and the mentioned disynaptic connections enable STN to produce downstream changes in the cerebellum and thus closing the loop [44]. Taken together, the role of cerebellar lobule VIIb in PD tremor is in line with the dimmer switch model and is further supported by evidence of grey matter atrophy in this lobule in tremor-dominant individuals with PD [45]. Exact tracing and topological mapping of cerebellar atrophy in association with tremor severity have proven to be challenging, as can be concluded from numerous but inconsistent reports of various cerebellar lobules being involved, including lobules IV, V, VI, VIIIa, VIIIb, crus I and the vermis [16, 18, 19, 46–48].

Importantly, the structural-clinical association revealed in this study is driven by kinetic tremor and not postural or rest tremor. Evidence from literature in terms of separating PD tremor types in the cerebellar structural analysis is sparse. So far, no significant volumetric association of cerebellar lobules with PD kinetic tremor has previously been reported. Of note, in essential tremor (ET) significant correlations between kinetic tremor and cerebellar lobule VIIb (among other lobules) volume have been reported [49, 50]. In comparison, PD rest tremor severity has been localized to lobule IV by Lopez et al. [18], and to lobule VIIIa with no significant results for kinetic or postural tremor severities [51]. The difference in findings could be due to different pathophysiology underlying distinct tremor manifestations in PD [42].

The volumetric association revealed in this study is exclusive to tremor and not to rigidity and bradykinesia severity. Also, it is solely observable for tremor of the hand and not for legs and jaw, or lips. Both of these findings are in line with recent studies that report cerebellar association only with MDS-UPDRS tremor scores and only for upper limbs [18, 51].

Furthermore, age exhibited no contribution to the main outcome whereas longer disease duration was significantly associated with lower tremor severity. Both of these factors could potentially be determinants of clinical features of PD [52], nevertheless, high variability in disease duration, as well as limited effect size, complicates further interpretation of such results [5].

The limited sample size brings further constraints to generalizing our findings. To address this issue and add robustness to our statistical analysis, LOOA was applied with high specificity at the cost of low sensitivity. We did not investigate structural differences specifically between subtypes of PD (TD vs. PIGD) due to insufficient number of participants belonging to each PD subtype in this study that could be grouped into corresponding balanced cohorts. Conducting regression models with small effect size would result in limited statistical power and the outcome would consequently be of less reliability [53]. For this reason, no further regression analysis was performed for subtypes of PD in the present study. Regardless, future studies are encouraged to recruit larger cohorts of participants with PD divided into age- and sex-matched groups based on PD subtypes, in order to enable concrete investigation of cerebellar volumetric associations with motor scores not only within but also between PD subtype cohorts. Longitudinal MDS-UPDRS and imaging data would bring even more robustness and enable observations of age and disease duration affecting the long-term development of volumetric pathologies in PD.

Taken together, our study recognizes smaller volume of lobule VIIb that could serve as a potential biological marker for tremor severity in PD, as defined by Miller et al. (41). This finding contributes to further enhancing the cerebellar morphological mapping of the tremor-related regions and networks. The identification of this structural feature may be of relevance for prognostic purposes in prospective longitudinal studies as well as for therapeutic trials. However, future studies in larger cohorts are required also taking advantage of higher-resolution MRI images (> 3 T), and finally adding special MRI sequences such as quantitative susceptibility mapping (QSM) to highlight fine structures such as the dentate nucleus.

### Supplementary Information

Below is the link to the electronic supplementary material.

Supplementary file1 (DOCX 35 KB)

## Data Availability

The data and material supporting the findings of this study are available upon reasonable request from the corresponding author.

## Footnotes

1 accessible via the following link: https://mds.movementdisorders.org/updrs/
