# Supplementary material for: Smaller Cerebellar Lobule VIIb is Associated with Tremor Severity in Parkinson’s Disease
Source: Cerebellum. 2023 Feb 20;23(2):355–62. doi: 10.1007/s12311-023-01532-6 (PMC10950956; doi:10.1007/s12311-023-01532-6)
Supplement: Supplementary file 1 — Supplementary file1 (DOCX 35 KB) [file 12311_2023_1532_MOESM1_ESM.docx]

Smaller cerebellar lobule VIIb is associated with tremor severity in Parkinson’s Disease

**Supplementary Material**

# **Descriptive Information on Cerebellar Volumes**

**Table S1.** descriptive statistics of cerebellar volumes

| **Cerebellar Region** | **Mean volume (cm^3^)** | **SD** | **Median [Min, Max] (cm^3^)** |
| --- | --- | --- | --- |
| Cerebellum | 136.82 | 15.59 | 135.85 [107.63, 185.46] |
| Lobule I-II | 0.10 | 0.04 | 0.09 [0.04, 0.28] |
| Lobule III | 1.43 | 0.29 | 1.40 [0.88, 2.28] |
| Lobule IV | 4.54 | 0.71 | 4.50 [2.50, 6.34] |
| Lobule V | 7.86 | 1.12 | 7.68 [6.13, 11.34] |
| Lobule VI | 17.89 | 3.38 | 17.89 [1.60, 26.61] |
| Crus I | 28.41 | 5.22 | 27.40 [19.33, 42.83] |
| Crus II | 17.58 | 2.99 | 17.24 [12.04, 24.40] |
| Lobule VIIb | 9.21 | 1.32 | 9.04 [6.86, 12.48] |
| Lobule VIIIa | 13.54 | 1.95 | 13.62 [9.88, 16.95] |
| Lobule VIIIb | 9.60 | 1.77 | 9.47 [6.31, 14.43] |
| Lobule IX | 7.86 | 1.50 | 7.74 [4.47, 11.71] |
| Lobule X | 1.58 | 0.28 | 1.55 [1.08, 2.36] |

**SD**: standard deviation

# **Association of Cerebellar Volumes with Bradykinesia-Rigidity Scores**

**Table S2.** Association between volumes of the cerebellum as well as cerebellar lobules and bradykinesia-rigidity (BR) score in Parkinson’s disease (PD). BR score has been extracted from MDS-UPDRS part III (items 3.3, 3.4, 3.5, 3.6, 3.7, and 3.8). Results of multiple linear regression models are presented and the primary outcome is demonstrated using the beta coefficient. The confidence interval is considered 95%. No significant association was found between cerebellar lobule volumes and BR score. Results are adjusted for age, sex, disease duration, and intercranial volume (ICV).

| **Cerebellar Region** | **Beta (coefficient)**  **(95% CI)** | **P-value** |
| --- | --- | --- |
| Cerebellum | 0.07 | 0.526 |
| Lobule I-II | -16.37 | 0.520 |
| Lobule III | 2.75 | 0.499 |
| Lobule IV | 1.34 | 0.443 |
| Lobule V | -0.81 | 0.454 |
| Lobule VI | -0.17 | 0.660 |
| Crus I | 0.38 | 0.167 |
| Crus II | 0.31 | 0.459 |
| Lobule VIIb | 0.04 | 0.967 |
| Lobule VIIIa | 0.29 | 0.656 |
| Lobule VIIIb | 0.52 | 0.514 |
| Lobule IX | -0.50 | 0.573 |
| Lobule X | 1.21 | 0.789 |

* p-value significant after leave-one-out (LOOA) analysis as well as FDR correction

# **Association of Cerebellar Volumes with Postural Instability and Gait Disorders Scores**

**Table S3.** Association between volumes of the cerebellum as well as cerebellar lobules and Postural instability and gait disorders (PIGD) score in Parkinson’s disease (PD). PIGD score has been extracted from MDS-UPDRS parts II and III (items 2.12, 2.13, 3.10, 3.11, 3.12). Results of multiple linear regression models are presented and the primary outcome is demonstrated using the beta coefficient. The confidence interval is considered 95%. No significant association was found between cerebellar lobule volumes and PIGD score. Results are adjusted for age, sex, disease duration, and intracranial volume (ICV).

| **Cerebellar Region** | **Beta (coefficient)**  **(95% CI)** | **P-value** |
| --- | --- | --- |
| Cerebellum | 0.01 | 0.862 |
| Lobule I-II | -4.36 | 0.738 |
| Lobule III | 1.72 | 0.410 |
| Lobule IV | 0.63 | 0.478 |
| Lobule V | -0.66 | 0.231 |
| Lobule VI | -0.28 | 0.146 |
| Crus I | 0.11 | 0.434 |
| Crus II | 0.24 | 0.274 |
| Lobule VIIb | 0.46 | 0.325 |
| Lobule VIIIa | -0.29 | 0.388 |
| Lobule VIIIb | -0.09 | 0.823 |
| Lobule IX | -0.48 | 0.295 |
| Lobule X | 3.50 | 0.127 |

* p-value significant after leave-one-out (LOOA) analysis as well as FDR correction

# **Association of Cerebellar Volumes with Distinct Tremor Types**

**Table S4.** Association between cerebellar lobule volumes and Parkinson’s disease (PD) postural tremor scores (extracted from MDS-UPDRS part III item 3.15). Outcome of the linear regression models reported as beta coefficient. The confidence interval is considered 95%. Results are adjusted for age, sex, and intracranial volume (ICV).

| **Cerebellar Region** | **Beta (coefficient)**  **(95% CI)** | **P-value** |
| --- | --- | --- |
| **Cerebellum** | -0.01 | 0.158 |
| Lobule I-II | -1.73 | 0.038 |
| Lobule III | -0.25 | 0.063 |
| Lobule IV | 0.00 | 0.981 |
| Lobule V | -0.02 | 0.526 |
| Lobule VI | 0.01 | 0.637 |
| Crus I | 0.00 | 0.591 |
| Crus II | -0.03 | 0.015 |
| Lobule VIIb | -0.08 | 0.005 |
| Lobule VIIIa | -0.02 | 0.275 |
| Lobule VIIIb | -0.02 | 0.437 |
| Lobule IX | -0.04 | 0.128 |
| Lobule X | -0.27 | 0.067 |

* p-value significant after leave-one-out (LOOA) analysis as well as FDR correction

**Table S5.** Association between cerebellar lobule volumes and Parkinson’s disease (PD) kinetic tremor scores (extracted from MDS-UPDRS part III item 3.16). Outcome of the linear regression models reported as beta coefficient. The confidence interval is considered 95%. Cerebellar lobules exhibiting significant association with tremor severity score are highlighted in bold. Results are adjusted for age, sex, and intracranial volume (ICV).

| **Cerebellar Region** | **Beta (coefficient)**  **(95% CI)** | **P-value** |
| --- | --- | --- |
| Cerebellum | -0.01 | 0.006 |
| Lobule I-II | 0.06 | 0.941 |
| Lobule III | -0.12 | 0.369 |
| Lobule IV | -0.09 | 0.095 |
| Lobule V | -0.05 | 0.148 |
| Lobule VI | -0.02 | 0.188 |
| Crus I | -0.01 | 0.363 |
| Crus II | -0.04 | 0.005 |
| **Lobule VIIb** | **-0.09** | **0.002** |
| Lobule VIIIa | -0.04 | 0.090 |
| Lobule VIIIb | -0.02 | 0.421 |
| Lobule IX | -0.02 | 0.418 |
| Lobule X | -0.22 | 0.139 |

* p-value significant after leave-one-out (LOOA) analysis as well as FDR correction

**Table S6.** Association between cerebellar lobule volumes and Parkinson’s disease (PD) rest tremor scores (extracted from MDS-UPDRS part III items 3.17 plus 3.18). Outcome of the linear regression models reported as beta coefficient. The confidence interval is considered 95%. Results are adjusted for age, sex, and intracranial volume (ICV).

| **Cerebellar Region** | **Beta (coefficient)**  **(95% CI)** | **P-value** |
| --- | --- | --- |
| Cerebellum | 0.00 | 0.877 |
| Lobule I-II | -3.10 | 0.006 |
| Lobule III | -0.41 | 0.027 |
| Lobule IV | 0.13 | 0.103 |
| Lobule V | 0.01 | 0.857 |
| Lobule VI | 0.01 | 0.623 |
| Crus I | 0.01 | 0.460 |
| Crus II | -0.01 | 0.789 |
| Lobule VIIb | -0.06 | 0.145 |
| Lobule VIIIa | 0.01 | 0.800 |
| Lobule VIIIb | 0.01 | 0.722 |
| Lobule IX | 0.02 | 0.573 |
| Lobule X | 0.15 | 0.468 |

** p-value significant after leave-one-out (LOOA) analysis as well as FDR correction*

# **Association of Cerebellar Volumes with Tremor in Upper Extremities**

According to MDS-UPDRS, “tremor score” consists of not only tremor observed in the upper extremities (hands) which are reflected in test items 2.10, 3.15a-b, 3.16a-b, 3.17a-b, and 3.18, but also lower extremities (legs) reflected in test items 3.17c and 3.17d, as well as jaw/lips tremor including score 3.17e. Consequently, the total tremor score is calculated from all these parameters. To address this point, additional analyses has been performed using the hand tremor severity only, meaning that the UPDRS leg and jaw/lips tremor scores were subtracted from the total tremor scores of each participant with PD and similar multiple linear regression models were utilized. The main results as reported in the manuscript are unchanged and reveal a significant correlation between the volume of lobule VIIb and hand tremor severity (P=0.005) as can be viewed in Table S4 below.

**Table S7.** Association between cerebellar lobule volumes and Parkinson’s disease (PD) tremor scores of the upper extremities. Outcome reported from the winning linear regression model via beta coefficient. The confidence interval is considered 95%. Cerebellar lobules exhibiting significant association with tremor severity score are highlighted in bold. Results are adjusted for age, sex, and intracranial volume (ICV).

| **Cerebellar Region** | **Beta (coefficient)**  **(95% CI)** | **P-value** |
| --- | --- | --- |
| Cerebellum | -0.01 | 0.227 |
| Lobule I-II | -2.75 | 0.021 |
| Lobule III | -0.36 | 0.059 |
| Lobule IV | 0.06 | 0.448 |
| Lobule V | -0.02 | 0.685 |
| Lobule VI | <0.01 | 0.828 |
| Crus I | 0.01 | 0.550 |
| Crus II | -0.03 | 0.113 |
| **Lobule VIIb** | **-0.12** | **0.005*** |
| Lobule VIIIa | -0.04 | 0.191 |
| Lobule VIIIb | -0.03 | 0.450 |
| Lobule IX | -0.04 | 0.301 |
| Lobule X | -0.09 | 0.676 |

* p-value significant after leave-one-out (LOOA) analysis as well as FDR correction
